# Two novel *ANK1* loss‐of‐function mutations in Chinese families with hereditary spherocytosis

**DOI:** 10.1111/jcmm.14343

**Published:** 2019-04-23

**Authors:** Lili Hao, Shanshan Li, Duan Ma, Shiyu Chen, Bowen Zhang, Deyong Xiao, Jin Zhang, Nan Jiang, Shayi Jiang, Jing Ma

**Affiliations:** ^1^ Department of Biochemistry and Molecular Biology, Key Laboratory of Metabolism and Molecular Medicine, Ministry of Education, School of Basic Medical Sciences Fudan University Shanghai China; ^2^ Shanghai Children's Hospital, Shanghai Jiao Tong University Shanghai China; ^3^ Pediatrics Research Institute Children's Hospital of Fudan University Shanghai China; ^4^ Research Center for Birth Defects, Institutes of Biomedical Sciences Fudan University Shanghai China; ^5^ Shanghai Key Lab of Birth Defect Children's Hospital of Fudan University Shanghai China

**Keywords:** *ANK1*, hereditary spherocytosis, in vitro experiment, mutation, NGS

## Abstract

Hereditary spherocytosis (HS) is the most common inherited haemolytic anaemia disorder. *ANK1* mutations account for most HS cases, but pathogenicity analysis and functional research have not been widely performed for these mutations. In this study, in order to confirm diagnosis, gene mutation was screened in two unrelated Chinese families with HS by a next‐generation sequencing (NGS) panel and then confirmed by Sanger sequencing. Two novel heterozygous mutations (c.C841T, p.R281X and c.T290G, p.L97R) of the *ANK1* gene were identified in the two families respectively. Then, the pathogenicity of the two new mutations and two previously reported *ANK1* mutations (c.C648G, p.Y216X and c.G424T, p.E142X) were studied by in vitro experiments. The four mutations increased the osmotic fragility of cells, reduced the stabilities of ANK1 proteins and prevented the protein from localizing to the plasma membrane and interacting with SPTB and SLC4A1. We classified these four mutations into disease‐causing mutations for HS. Thus, conducting the same mutation test and providing genetic counselling for the two families were meaningful and significant. Moreover, the identification of two novel mutations enriches the *ANK1* mutation database, especially in China.

## 1 INTRODUCTION

Hereditary spherocytosis (HS), one of the most common monogenic haemolytic anaemia diseases, is characterized by spherical‐shaped erythrocytes in the peripheral blood of patients. Hereditary spherocytosis is prevalent worldwide, especially in the Northern European population, with an incidence rate of 1:2000.^1^, ^2^, ^3^ To date, no exact epidemiological data related to HS in China are available, and the estimated prevalence is 1.27/100 000 in males and 1.49/100 000 in females, which was reported by Wang et al only.^4^

Hereditary spherocytosis belongs to a heterogeneous group of erythrocyte membrane disorders caused by gene mutations; these conditions range in severity from asymptomatic to severe transfusion‐dependent states with several main clinical manifestations, including anaemia, jaundice, splenomegaly and cholelithiasis.^5^ In the clinic, making an exact diagnosis of asymptomatic or atypical cases is relatively difficult based on clinical symptoms, family history and haematologic parameter tests alone.^6^, ^7^ Therefore, molecular detection is particularly important for the clinical diagnosis of HS.

Hereditary spherocytosis is caused by mutations of erythrocyte membrane‐related genes, including *ANK1* (ankyrin 1; OMIM 612641), *SPTB* (spectrin, beta, erythrocytic; OMIM 182870), *SPTA1* (spectrin, alpha, erythrocytic 1; OMIM 182860), *SLC4A1* (solute carrier family 4, member 1; OMIM 109270) and *EPB42* (protein 4.2, erythrocytic; OMIM 177070), following an autosomal dominant (AD) and autosomal recessive inheritance mode.^8^ Mutations in these genes usually produce decreased surface area per unit volume of erythrocytes or dysfunction of the erythrocyte membrane, leading to the detachment of the lipid bilayer from the spectrin‐based cytoskeleton. Therefore, abnormal erythrocytes become spherocytes with increased osmotic fragility and are easily destroyed by the spleen, consequently resulting in haemolytic anaemia.^9^ Mutations in the *ANK1* gene can explain approximately half of all cases with HS. Additionally, 80%‐85% of *ANK1* mutations are AD inherited.^10^

The *ANK1* gene is located at 8p11.21.^11^ *ANK1* contains 43 exons, and the cDNA is 8 300 bp in length, coding for erythroid ankyrin 1 protein of 1 880 amino acids with three main structural domains, an N‐terminal membrane‐binding domain, a central spectrin‐binding domain and a C‐terminal regulatory domain, which is the least conserved and subject to variation.^12^ ANK1 protein serves as the key link between the SLC4A1 (band 3) complex on the lipid bilayer to the erythrocyte cytoskeleton and confers vertical stability and reversible deformability to the erythrocyte membrane.^13^ A mouse model with truncated mutations lacking the spectrin‐binding and C‐terminal regulatory domains of Ank1 manifested severe HS.^8^

To date, a total of 89 *ANK1* mutations have been reported in the Human Gene Mutation Database (registration required, http://www.hgmd.cf.ac.uk, last accessed 24 May 2018), including 38 missense or nonsense mutations. Although mutations in *ANK1* are common in HS, the pathogenic mechanism of *ANK1* mutations is not entirely understood.

In this study, two novel mutations of *ANK1* were found in two unrelated Chinese families with HS and predicted to be disease causing. Then, we carried out functional research in vitro to further confirm the pathogenicity of four *ANK1* mutations, including the two novel mutations and two previously reported mutations (c.G424T, p.E142X and c.C648G, p.Y216X) in a Chinese population.^7^

## 2 MATERIALS AND METHODS

### 2.1 Pedigrees and ethical statement

**Figure 1:**
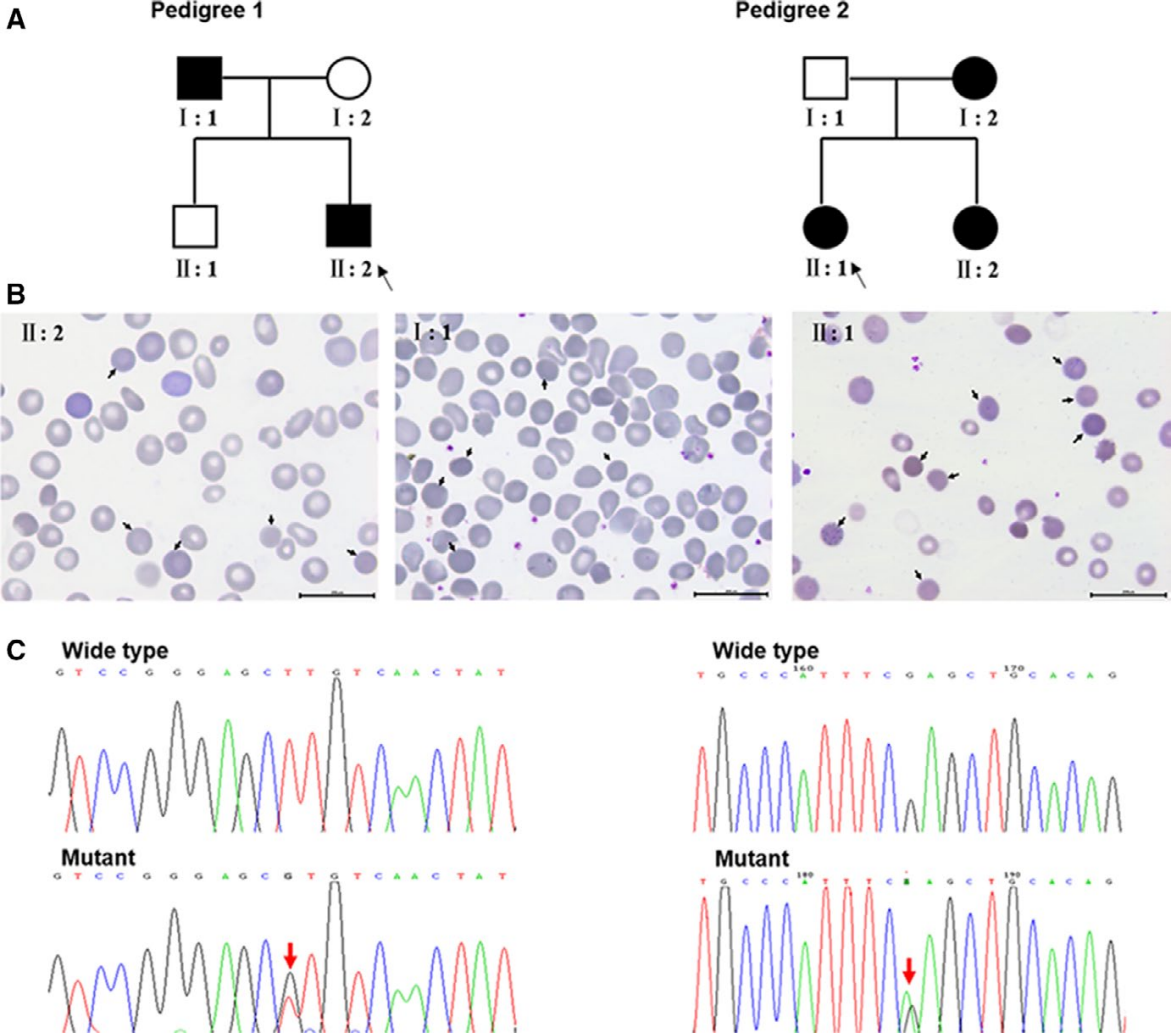
Two unrelated Chinese families with HS‐associated *ANK1* mutations. (A) Pedigrees: open symbols indicate unaffected individuals; filled black symbols indicate affected individuals; and black arrows indicate probands. The left is pedigree 1; member I: 1 and II: 2 carry the novel *ANK1* mutation (c.T290G, p.L97R), and I: 2 and II: 1 do not have this mutation. The right is pedigree 2; the novel *ANK1* mutation (c.C841T, p.R281X) was found in member II: 1, II: 2, I: 1 and was not identified in I: 2. (B) Peripheral blood smears of patients in two pedigrees. Spherocytes are observed in the blood films of the patients in two pedigrees (pedigree 1: member I: 1 and II: 2; pedigree 2: member II: 1) which are indicated by black arrows. (C) Partial DNA sequences containing the two novel heterozygous *ANK1* mutations. The upper panels show the wild‐type sequences, and in the lower panels, red arrows indicate the mutation sites

Two unrelated Chinese nuclear families with HS were recruited into the study (Figure 1A). In pedigree 1, the male newborn (II: 2) had suspected HS with severe anaemia, jaundice and splenomegaly 3 months after birth and received several blood transfusions. His father (I: 1) underwent splenectomy because of HS at 1 year old. His mother (I: 2) and elder brother (II: 2) were non‐symptomatic. In pedigree 2, the proband (II: 1) was a 4‐year‐old girl with suspected HS who presented with severe haemolytic anaemia. Her mother (I: 2) underwent splenectomy due to HS when she was 10 years old. Her father (I: 1) showed abnormalities in the erythrocyte membrane, and her younger sister (II: 2) showed mild anaemia. In the two pedigrees, the probands underwent a series of detailed blood tests. Glucose‐6‐phosphate dehydrogenase activity was normal. The results of an autoimmune antibody test were negative. Spherocytes were observed in a peripheral blood smear (Figure 1B), and osmotic fragility was increased (Table 1). The other available blood test data are summarized in Table 1. Then, whole blood samples of the proband and three other members in her/his nuclear family were collected for gene screening by NGS Confirmation testing, such as specific site analysis, was performed by Sanger sequencing. The research was formally approved by the Ethics Committee of Shanghai Children's Hospital. All eight participants underwent pre‐test counselling; during counselling, they were informed about the significance of gene screening, and signed written informed consent conforming to the tenets of the Declaration of Helsinki (1983 Revision). All procedures were performed in accordance with the approved guidelines.

**Table 1:** Laboratory test results

Tests	Pedigree 1		Pedigree 2		References
	II: 2	I: 1	II: 2	II: 1	
RBC (×10^12^/L)	2.22	5.14	1.67	3.35	4.00‐5.50
Hb (g/L)	60.00	168.00	48.00	95.00	110.00‐160.00
MCV	76.00	88.90	91.60	77.90	73.00‐100.00
MCH (pg)	27.00	32.70	28.70	28.00	27.00‐32.00
MCHC (g/L)	355.00	368.00	314.00	360.00	320.00‐410.00
RDW	21.40	12.90	31.80	NA	11.00‐16.00
PCV (%)	13.04	45.70	15.30	26.40	34.00‐48.00
Reticulocyte	7.57	2.48	8.55	8.55	0.50‐1.50
T‐Bil (μmol/L)	53.53	NA	43.40	NA	3.40‐17.10
D‐Bil (μmol/L)	9.40	NA	9.00	NA	0‐6.80
Haemolysis begins	0.64	0.60	0.62	0.50	0.38‐0.40
Haemolysis complete	0.44	0.44	0.44	0.36	0.30‐0.34

Abbreviations: D‐Bil, direct bilirubin; MCH, mean corpuscular haemoglobin; MCHC, mean corpuscular haemoglobin concentration; MCV, mean corpuscular volume; NA, not available; PCV, packed cell volume; RBC, red blood cell; RDW, red blood cell distribution width; T‐Bil, total bilirubin.

### 2.2 Genetic analysis

A NGS panel of 700 genes associated with hereditary haematological and immunodeficiency diseases was used for genetic testing. The probes covered all the exons and corresponding splice junctions of genes. DNA samples were extracted from whole blood using a QIAamp DNA Blood Mini Kit (Qiagen, Hilden, Germany). Sequencing libraries were prepared using an Ion Plus Fragment Library Kit (Life Technologies, Carlsbad, CA). Sequencing was accomplished on an ion Torrent PGM™ platform according to the sequencing protocol. Sequences were aligned to human genome reference version hg19, and variants were filtered using several common variant databases, including NCBI dbSNP (https://www.ncbi.nlm.nih.gov/SNP/), HapMap^14^ and the 1000 Human Genomes Project.^15^ The pathogenicity prediction of identified variants was made by SIFT^16^ (http://sift.jcvi.org/), PolyPhen‐2^17^ (http://genetics.bwh.harvard.edu/pph2/) and MutationTaster^18^ (http://www.mutationtaster.org/). Conservation analysis was performed by ClustalX 2.0.11 programs^19^ to compare the human wild‐type ANK1 protein sequence (NP_000028.3) with orthologues from *Pan troglodytes*, *Physeter catodon*, *Macaca mulatta*, *Mus musculus*, *Gallus gallus*, *Danio rerio* and *Xenopus tropicalis*. Through this screening, we identified two novel mutations of *ANK1* in the two families with HS respectively. For mutation confirmation, the amplicons were subjected to bidirectional automated DNA sequencing by an ABI Prism 3730XL Genetic Analyzer (Applied Biosystems Inc, Foster City, CA) according to the manufacturer's instructions. The primers were as follows: ANK1‐M1 (T290G)‐F: 5'‐CTTCCCCCATGTGTTTCAGA‐3', ANK1‐M1 (T290G)‐R: 5'‐TTACTTCTGTGGCTACATTCTGG‐3'; ANK1‐M2 (C841T)‐F: 5'‐CTCCTACGTGATAGGCCTTGT‐3', ANK1‐M2 (C841T)‐R: 5'‐GTGCAGTGGGAGGCAATAGT‐3'.

### 2.3 Pathogenicity prediction

ANK1 structural changes caused by the mutations were predicted and analysed by SWISS‐MODEL (http://swissmodel.expasy.org).

### 2.4 Preparation of mutant constructs

A plasmid containing full‐length *ANK1* cDNA (BC030957) was purchased from abmgood (Applied Biological Materials, Inc, Richmond, Canada). Wild‐type *ANK1* was cloned into the pCDNA3.1(+)‐3ΧFlag‐C expression vector, and 5'‐ATG GACTACAAGGACGACGATGACAAG‐3' (FLAG) was inserted before the initiation codon (FLAG‐WT‐ANK1). *ANK1* mutants (FLAG‐M1‐ANK1, FLAG‐M2‐ANK1, FLAG‐M3 (G424T)‐ANK1 and FLAG‐M4 (C648G)‐ANK1) were obtained by a KOD‐Plus Mutagenesis Kit (TOYOBO, Osaka, Japan). The primers used were as follows:

ANK1‐M1‐F: 5'‐GTGTCAACTATGGAGCCAACGTCA‐3'

ANK1‐M1‐R: 5'‐GCTCCCGGACCACCTCATCCT‐3'

ANK1‐M2‐F: 5'‐TAAGACGGCTTCACGCCTCTG‐3'

ANK1‐M2‐R: 5'‐TGTGGCTACATTCTGGTTAGCTCCA‐3'

ANK1‐M3‐F: 5'‐GGAGAACCTCAACGTGGCCCAGTTGCTCCTCA‐3'

ANK1‐M3‐R: 5'‐TAGTGAGCCGCAATGTGCAGGGGCGTGAATC‐3'

ANK1‐M4‐F: 5'‐TGAAATGGGCACGTGCGAATCT‐3'

ANK1‐M4‐R: 5'‐AGCTGCACAGTGGAGAGGTGTCA‐3'

All expression constructs were validated by Sanger sequencing.

### 2.5 Cell culture and transfection

Human embryonic kidney 293T (HEK293T) cells were grown and maintained in Dulbecco's Modified Eagle's Medium with 10% foetal bovine serum (FBS) and 1% Pen‐Strep antibiotics at 37°C and 5% CO_2_. Before transfection, cells were seeded in 6‐well culture dishes at a density of 5 × 10^5^ cells/well. HEK293T cells were transiently transfected with FLAG‐WT‐ANK1, FLAG‐M1‐ANK1, FLAG‐M2‐ANK1, FLAG‐M3‐ANK1 and FLAG‐M4‐ANK1 separately (a total of 1.2 μg of each plasmid DNA) by Viafect transfection agent (Promega, Madison, WI). Cells were harvested 48 hours after transfection.

### 2.6 Osmotic fragility test

Transfected HEK293T cells were digested by trypsin, washed with phosphate‐buffered saline (PBS) and centrifuged at 1000 rpm for 5 minutes. After the supernatant was discarded, 1 × 10^6^ cells were suspended in 300‐μL hypotonic sodium chloride (NaCl) solution with different concentrations (0%‐0.85%) for 10 minutes. The appropriate concentrations were selected by observing cell morphology (beginning to rupture and completely rupturing) under a microscope. Then, the number of living cells (LCN) in a 10‐μL cell suspension was measured using a Countess II FL Automated Cell Counter (Invitrogen, Carlsbad, CA). The rate of cell rupture in NaCl solution with different concentrations was used to evaluate the osmotic fragility of HEK293T cells and calculated by LCN. The calculations are as follows:

$$\text{Rate}\;\;\text{of}\;\text{cell}\;\text{rupture}\;=\frac{\text{LCN}\;\;\text{under}\;\text{normal}\;\text{line}\;\left(0.85\text{\textbackslash{}\%}\;\right)-\text{LCN}\;\text{under}\;\text{different}\;\text{NaCl}\;\text{solution}}{\text{LCN}\;\text{under}\;\text{normal}\;\text{saline}\;\left(0.85\text{\textbackslash{}\%}\;\right)}\times 100\text{\textbackslash{}\%}\;$$

### 2.7 RNA extraction and quantitative real‑time polymerase chain reaction

Total RNA of transfected HEK293T cells was extracted using TRIzol reagent (Invitrogen) and reverse transcribed to cDNA with a Prime Script RT Reagent Kit (TaKaRa, Dalian, China). Quantitative real‑time polymerase chain reaction (qPCR) was conducted by SYBR Premix Ex Taq™ (Takara) on a Roche 480 plus system (Roche, Mannheim, Germany). The primers are as follows:

ANK1‐F: 5'‐AGCCGGACCTGATAGAGG‐3',

ANK1‐R: 5'‐CATGGTCTTTGTAGTCGAAGGG‐3'

GAPDH‐F: 5'‐CACCCACTCCTCCACCTTTG‐3',

GAPDH‐R 5'‐ACCACCCTGTTGCTGTAGCC‐3'

Gene expression was normalized to GAPDH and analysed according to the relative quantification method (2^−ΔΔCt^). Three independent experiments were performed.

### 2.8 Western blotting

Transfected HEK293T cells were lysed in RIPA buffer (Calbiochem/EMD Millipore, Billerica, MA) with protease inhibitor cocktail (Roche). Protein determination was performed by a BCA Protein Assay Kit (Pierce, Rockford, IL). To detect the expression of FLAG‐WT‐ANK1, FLAG‐M1‐ANK1, FLAG‐M2‐ANK1, FLAG‐M3‐ANK1 and FLAG‐M4‐ANK1 in transfected HEK293T cells, we separated soluble proteins by 4%‐20% SDS‐PAGE and transferred the proteins to a polyvinylidene fluoride (PVDF) membrane where FLAG‐WT‐ANK1 ran at a size of ~250 kDa. Membranes were blocked in 10% milk, incubated with primary FLAG antibody (1:3000 dilution, M20008S, Abmart, Shanghai, China) diluted in 3% bovine serum albumin, washed and incubated with secondary HRP‐conjugated antibody (anti‐mouse, 1:2000 dilution, SA00001‐1, Proteintech, Rosemont, IL) diluted in 10% milk. SuperSignal™ West Pico PLUS Chemiluminescent Substrate (Thermo Fisher, Rockford, IL) was used to visualize proteins on X‐ray film.

### 2.9 Immunofluorescence

Transfected cells grown on cover slips to 70%‐80% confluence were fixed in 4% paraformaldehyde, then underwent permeation using PBS with 0.5% Triton and were blocked with 3% FBS in PBS‐Triton. Cells were incubated with anti‐FLAG antibody (1:200, Abmart) and anti‐Calnexin antibody (1:50, Proteintech) overnight at 4°C. Alexa Fluorescence series secondary antibodies (Life Technologies, Oregon) were used. Finally, the cells were fixed in the ProLong™ Diamond Antifade Mountant (Life Technologies) and then photographed by a Leica SP8 confocal microscope.

### 2.10 Cell surface biotinylation

The transfected HEK293T cells grown in 10‐cm culture dishes were washed three times and suspended with ice‐cold PBS (pH 8.0). Cells were treated with 1 mL of 1 mg/mL EZ‐Link NHS‐SS‐Biotin (Pierce, Rockford, IL) in PBS (pH 8.0) for 30 minutes at 4°C to biotinylate the cell surface and rinsed in PBS with 100‐mM glycine to quench any unreacted reagent. Then, the cells were lysed with RIPA buffer (Millipore) containing protease inhibitors. An aliquot of the lysate was saved for western blot analyses. ImmunoPure immobilized streptavidin beads 100 μL (Pierce) were added to the lysate overnight at 4°C to bind the biotinylated proteins. The supernatant was removed and an aliquot was saved for western blot analyses. The streptavidin beads were washed three times with RIPA buffer. Samples were solubilized in 2 × SDS buffer and analysed by 4%‐20% SDS‐PAGE. The transfection dose of all vectors was adjusted so that immunoreactivity of all samples could be showed on the PVDF membrane. The amount of ANK1‐FLAG proteins located on plasma membrane was normalized to the total amount of ANK1‐FLAG expressed in the cells.

### 2.11 Co‐immunoprecipitation

Human embryonic kidney 293T cells transiently transfected with wild‐type or mutant ANK1‐FLAG constructs were dissolved in RIPA buffer (Millipore) with protease inhibitor cocktail (Roche). Cell lysates were centrifuged at 12 000 rpm for 25 minutes at 4°C to remove insoluble material. Then supernatant was incubated with an anti‐FLAG affinity gel (Biotool, Switzerland) overnight at 4°C. Interactions between wild‐type or mutant ANK1 protein with two erythrocyte membrane proteins (SPTB and SLC4A1) were detected by western blot analyses with anti‐SPTB (1:800 dilution, ab129065, Abcam), anti‐SLC4A1 (1:800 dilution, ab108414, Abcam, Cambridge, UK) and anti‐FLAG (Abmart).

### 2.12 Statistical analysis

All statistical analyses were performed by paired two‐tailed Student's *t* test with GraphPad Software (La Jolla, CA) or Mantel‐Haenszel chi‐squared test (Biostatistics Services, IUSM).

All experiments were repeated more than twice.

## 3 RESULTS

### 3.1 Characterization of ANK1 mutations in two families with HS

**Figure 2:**
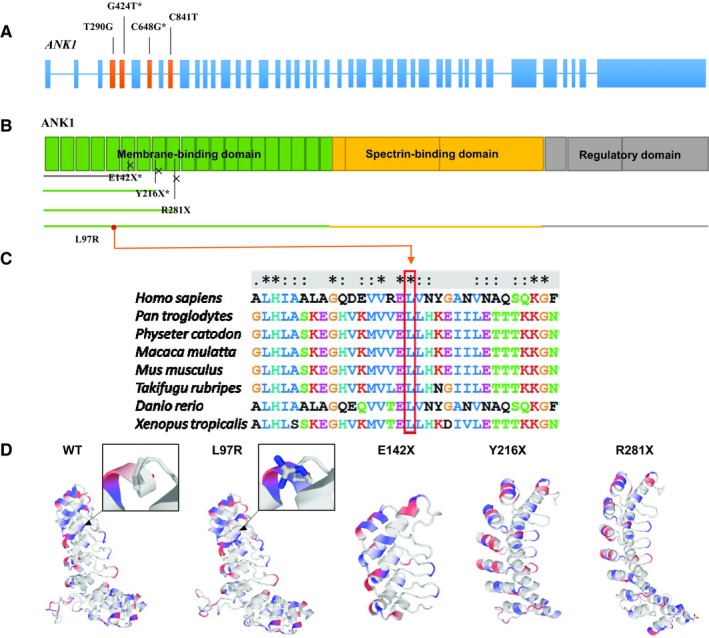
The pathogenicity of four ANK1 mutations predicted by bioinformatic analysis. (A) The structure of the *ANK1* gene. The whole gene has 43 exons. Four mutations (c.T290G, c.G424T, c.C648G, c.C841T) identified in the *ANK1* gene are in exons 4, 5, 7 and 9 respectively. (B) The ANK1 protein. Four mutations all occur in the membrane‐binding domain. Crosses indicate the truncation point caused by the three nonsense mutations. All the truncated proteins lack the spectrin‐binding domain, regulatory domain and part of the membrane‐binding domain. (C) Conservative analysis of the mutation site. The comparison results show that the Leu residue at 97, the missense mutation in the ANK1 protein, is well conserved across *Homo sapiens, Pan troglodytes, Physeter catodon, Macaca mulatta, Mus musculus, Takifugu rubripes, Danio rerio* and* Xenopus tropicalis*. (D) Structural change prediction of ANK1 mutants. The three‐dimensional structure of the ANK1 membrane‐binding domain (1‐827 amino acids) before and after mutation was predicted by SWISS‐MODEL. In the black box, the local structures of the site where mutation p.L97R occurs are partially enlarged; blue indicates the positively charged amino acid after mutation. Three‐dimensional structure of p.E142X, p.Y216X and p.R281X mutants indicates the incomplete structure of the domain because of the early terminated translation

In pedigree 1, a novel heterozygous mutation (c.T290G, p.L97R) in *ANK1* was detected in two patients (II: 2 and I: 1), while two other unaffected members (I: 2 and II: 1) had wild‐type *ANK1* alleles (Figure 1C). This missense mutation is located at exon 4 in the N‐terminal membrane‐binding domain and is highly conserved (Figure 2A and C). In pedigree 2, a novel heterozygous nonsense mutation in *ANK1* (c.C841T, p.R281X) was identified in patients (II: 1, II: 2 and I: 2), but I: 1 did not carry it (Figure 1C). The mutation is located in exon 9 of the N‐terminal membrane‐binding domain (Figure 2A and B). This mutation produced a truncated protein with a shortened membrane‐binding domain and without a spectrin‐binding domain as well as a regulatory domain (Figure 2B). Both novel mutations were predicted to be disease causing (Table 2).

**Table 2:** The information of the four mutations identified in ANK1

Nucleotide	Amino acid	Zygosity	Prediction information			ExAC or 1000G
			SIFT	Polyphen2	Mutation taster	
c.T290G	p.L97R	Hete	Damaging (0)	Probably damaging (1.00)	Disease causing (102)	New
c.C841T	p.R281X	Hete	—	—	Disease causing (6.0)	New

Note

The pathogenicity was tested using the bioinformatics software SIFT, PolyPhen2 and MutationTaster.

Abbreviations: c, variation at cDNA level; ExAC, Exome Aggregation Consortium; Hete, heterozygote; p, variation at protein level; X, Stop codon.

### 3.2 The predicted effect of four mutations on protein structure

The results of structure predictions showed that mutation p.L97R led to the change of non‐polar leucine to positively charged arginine at amino acid 97, thus altering the 3D structure of the N‐terminal membrane‐binding domain (Figure 2D). The other three nonsense mutations resulted in truncated proteins, including the incomplete N‐terminal membrane‐binding domain (Figure 2D). These changes might influence the combination of ANK1 and SLC4A1 and can lead to the non‐union of ANK1 with SPTB.

### 3.3 ANK1 mutations caused higher cell osmotic fragility

**Figure 3:**
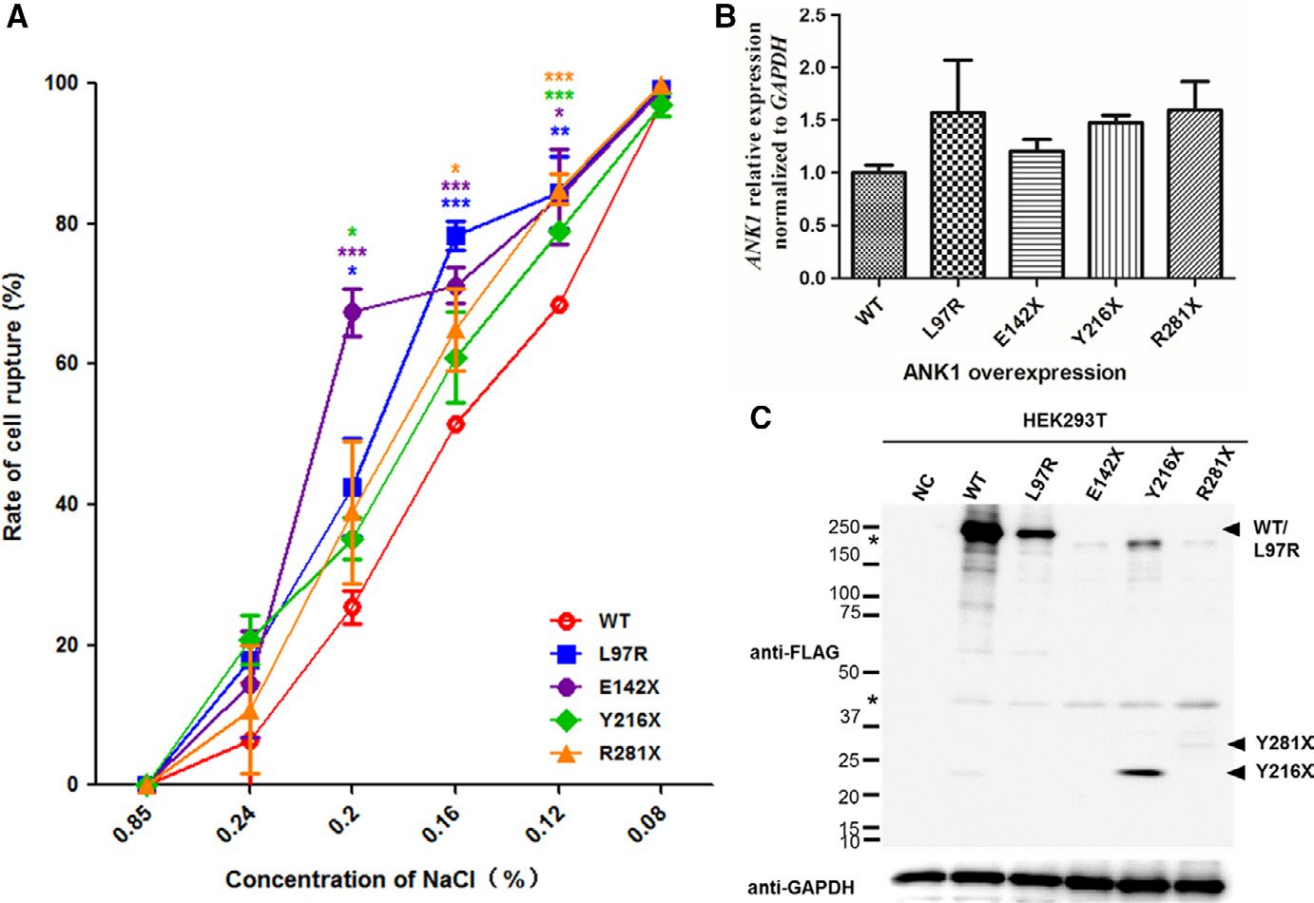
(A), Cell osmotic fragility test of human embryonic kidney 293T (HEK293T) cells transfected with FLAG‐WT‐ANK1 or FLAG‐M‐ANK1. The detection and calculation results show that the rate of cell rupture is higher when all mutant cells compared to wild‐type cells in NaCl solution within the concentration interval of 0.08%‐0.85%. Data are presented as the means ± SE (n = 3). *P < 0.05, **P < 0.01 and ***P < 0.001 when compared with wild‐type cells in every NaCl solution. (B, C) quantitative real‑time polymerase chain reaction (qPCR) and western blot analyses of HEK293T cells expressing FLAG‐WT‐ANK1 and FLAG‐M‐ANK1. (B) qPCR indicates that the four mutations do not affect the mRNA expression level of *ANK1.* The values represent the average over four experiments. (C) The results of anti‐FLAG western blot analyses show that L97R, Y216X and R281X proteins are expressed more weakly in HEK293T cells compared to wild‐type protein and the expression of E142X protein could not be detected. The asterisks indicate non‐specific or protein degradation product

Through a preliminary test, the number of broken HEK293 cells was found to be significantly increased when the concentration of NaCl was less than 0.24%, and almost all cells were completely broken when the concentration was as low as 0.08%. Therefore, we compared the change of cell rupture rate among wild‐type and mutant cells in NaCl solution between 0.08% and isotonic concentration of 0.85% (0.08%, 0.12%, 0.16%, 0.2%, 0.24% and 0.85%). The detection and calculation results showed that the rate of cell rupture with *ANK1* mutants was higher than that with wild‐type protein at every concentration of NaCl (Figure 3A). These results indicated that cell rupture occurred more easily in *ANK1* mutant cells, thus leading to higher cell osmotic fragility.

### 3.4 Protein stabilities were decreased in ANK1 mutants

To determine the pathogenicity of different *ANK1* mutations, we successfully overexpressed wild‐type and four mutant proteins in HEK293T cells. qPCR showed no differences in the transfection efficiency of different plasmids, and the mutations did not affect the mRNA expression level of *ANK1* (Figure 3B). However, western blot analyses revealed that the expression of all four mutants was weaker than that of wild‐type. In particular, truncated mutant p.E142X led to the total degradation of ANK1 (Figure 3C).

### 3.5 ANK1 mutants had defect in being located on the plasma membrane

**Figure 4:**
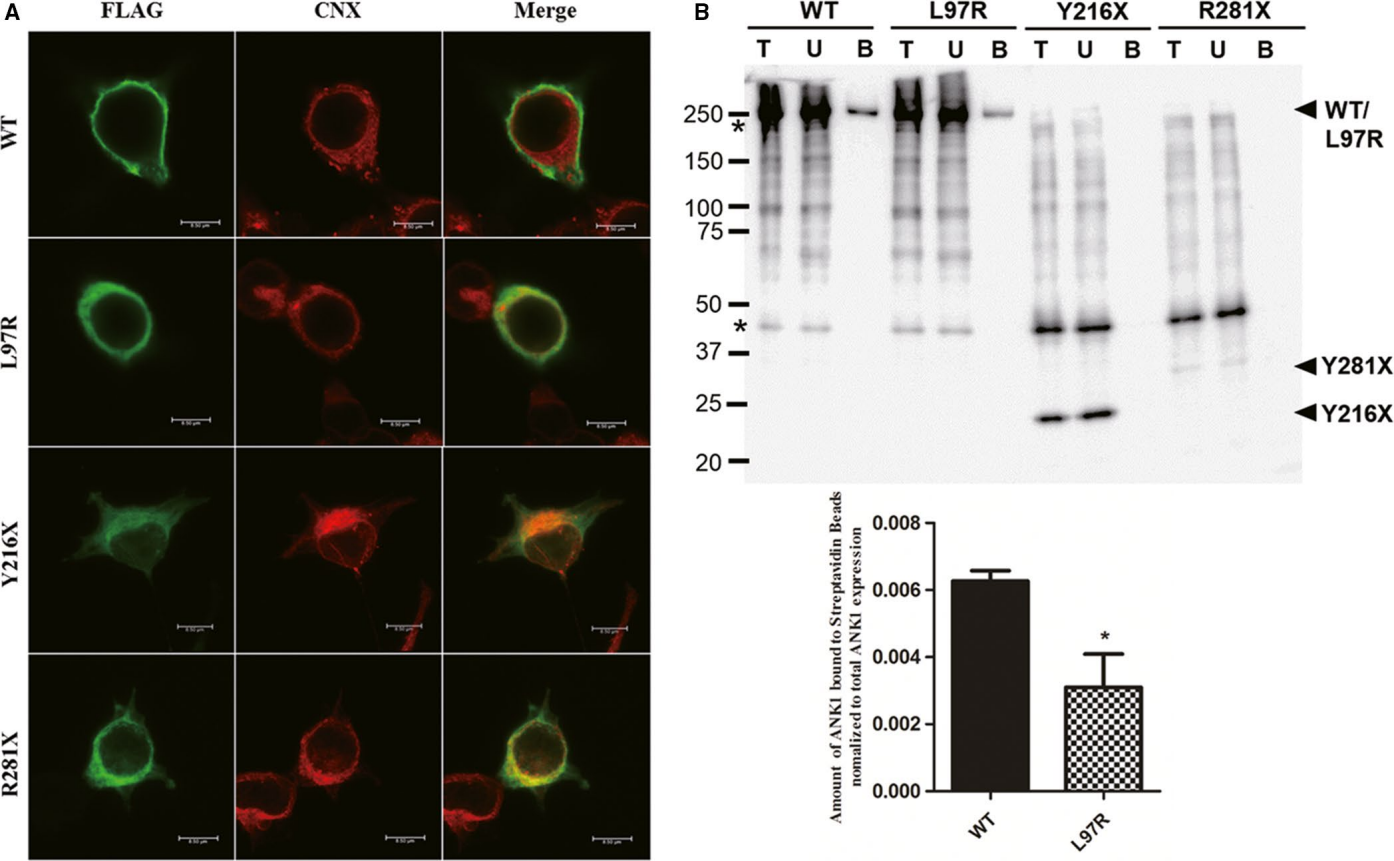
ANK1 mutants failed to localize to the plasma membrane. (A) Immunofluorescence of human embryonic kidney 293T (HEK293T) cells expressing FLAG‐WT‐ANK1 and FLAG‐M‐ANK1 (p.R281X, p.L97R and p.Y216X). Green fluorescence results from the FLAG fusions, and red fluorescence indicates the location of endoplasmic reticulum (ER) marked by Calnexin. The merged images show that the mutant ANK1 proteins are likely to be primarily retained in the ER rather than being targeted to the plasma membrane. (B) Cell surface biotinylation assay of wild‐type and mutant ANK1 in transfected HEK293T cells. Lane T: the amount of total ANK1 expressed. Lane U: the amount of ANK1 not bound to the streptavidin beads. Lane B: and the amount of ANK1 bound to the streptavidin beads. The asterisks indicate non‐specific or protein degradation product. The immunoblot results display that the amount of all the mutants is relatively less than the wild‐type ANK1 combined with the cellular membrane and two mutants Y216X and R281X are hardly bound

Mutations can affect the processing and structure of the protein, which may alter the cellular localization of protein. Thus, the subcellular localization of mutant ANK1 proteins was determined using Immunofluorescence (IF). Immunofluorescence indicated that wild‐type ANK1 was localized to the plasma membrane of HEK293T, while three mutants (L97R, Y216X and R281X) were mainly localized to the endoplasmic reticulum (ER) (Figure 4A). Cell surface biotinylation assay was performed to determine the amount of wild‐type and mutant ANK1‐FLAG proteins bound by the plasma membrane of transfected HEK293T cells. The results indicated that the amount of wild‐type ANK1‐FLAG was relatively more than all the mutants combined with the plasma membrane. Especially, the binding signals of the two ANK1 mutants Y216X and R281X with plasma membrane were almost undetectable even when the exposure intensity was elevated (Figure 4B). The mutant ANK1 proteins were likely to be primarily retained in the ER rather than being targeted to the plasma membrane, thus they lost their function as anchoring protein.

### 3.6 Mutations abolished ANK1 interaction with SPTB and SLC4A1

**Figure 5:**
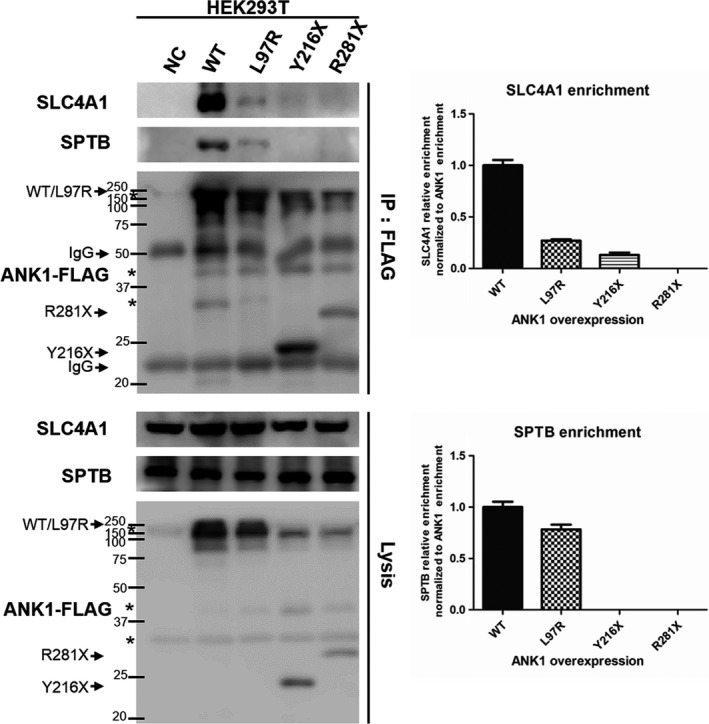
Co‐immunoprecipitation (Co‐IP) of wild‐type and mutant ANK1 with SPTB/SLC4A1 in human embryonic kidney 293T (HEK293T) cells. Co‐IP analysis of physiologically relevant ANK1‐SLC4A1/SPTB interactions in HEK293T cells using FLAG antibodies. None or little SCLC4A1/SPTB was captured when ANK1 was mutated in the three sites. The asterisks indicate non‐specific or protein degradation product. Semi‐quantitative analysis of the captured amount of SPTB/SLC4A1 relative to the amount of immunoprecipitated ANK1 was performed by greyscale scanning of strips. Data are the mean values of duplicate samples

The four mutations occurred in the N‐terminal membrane protein binding domain, and they failed to be transported to the plasma membrane, which indicated their combination with membrane protein SLC4A1 were impaired. Thus, the interaction between ANK1 mutants (L97R, Y216X and R281X) with SLC4A1 and another ANK1 major binding protein SPTB, was detected by co‐immunoprecipitation. The biochemical interactions between two mutants (Y216X and R281X) and SLC4A1/SPTB were greatly abolished in HEK293T cells. The L97R mutant had a normal interaction with SPTB and an attenuated interaction with SLC4A1, while wild‐type ANK1 could interact with the two proteins (Figure 5). Therefore, ANK1 mutants could not anchor SLC4A1 to the SPTB due to their inability to be localized on the plasma membrane.

## 4 DISCUSSION

Hereditary spherocytosis is a common inherited erythrocyte membrane disorder, and the clinical symptoms are variable even within one family.^7^, ^13^, ^20^ Cases with atypical clinical phenotypes or without family history defy diagnosis. The molecular genetic analysis of the proband and as many other family members as possible would be helpful to confirm the diagnosis, especially for situations in which the clinical phenotype of the proband is more severe than that of other affected members in the family.^9^ Mutations in *ANK1* are a common genetic cause of HS. More than half of HS patients bear *ANK1* mutations in many countries, such as the USA, Europe and South Korea, but the prevalence of *ANK1* mutations in China is unclear.

For a long time, the conventional mutation detection method for *ANK1* genes has been Sanger sequencing. However, the cost of direct sequencing is high because of the large size of the *ANK1* gene, its high‐allelic heterogeneity and the lack of hot‐spot mutations. Currently, the development of NGS technology provides a valid way to find the exact genetic cause of diseases. NGS could be rapider, more thorough and less expensive than Sanger sequencing, especially for the detection of large genes.^21^ As NGS is used more widely in clinical practice, an increasing number of mutations in *ANK1* have been identified as the genetic causes of HS, but the pathogenesis of these mutations has seldom been confirmed.

Here, we successfully identified two novel mutations of *ANK1* in two unrelated HS Chinese families, respectively, by a NGS panel. To fully identify the pathogenesis of ANK1 mutations in Chinese population, we also performed a functional study of two novel ANK1 mutations (c.T290G, p.L97R and c.C841T, p.R281X) we found and another two ANK1 mutations (c.G424T, p.E142X and c.C648G, p.Y216X) that have been previously reported.

Among the four mutations, three (c.G424T, p.E142X; c.C648G, p.Y216X and c.C841T, p.R281X) are nonsense mutations, and one (c.T290G, p.L97R) is a missense mutation. The three nonsense mutations produced truncated ANK1 proteins, which exhibited loss of the spectrin‐binding domain, regulatory domain and part of the membrane‐binding domain. Spectrin is the major constituent of the cytoskeletal network underlying the erythrocyte plasma membrane and associates with band 4.1 and actin to form the cytoskeletal superstructure of the erythrocyte plasma membrane. SLC4A1 is the major integral membrane glycoprotein of the erythrocyte membrane and is responsible for normal flexibility and stability of the erythrocyte membrane and for normal erythrocyte shape via interactions between its cytoplasmic domain and cytoskeletal proteins, glycolytic enzymes and haemoglobin.^22^ The 55 kDa regulatory domain of ANK1 is involved in regulating its association with SPTB and SLC4A1.^23^, ^24^ As the key to maintaining the shape of erythrocytes, all three domains of ANK1 are essential for appropriate function and assembly of the erythrocyte membranous‐cytoskeletal network. According to previous reports, ANK1 mutations, particularly nonsense and frameshift mutations carried by HS patients, could decrease the expression of ANK1.^25^, ^26^ Based on our in vivo experiments, the E142X mutant was completely degraded and non‐functional, and the expression of the other three mutants was also reduced. What's more, the ER retention of the misfolded ANK1 mutants (L97R, Y216X and R281X) was observed, which may lead to the degradation of the protein.^27^ The SPTB‐ANK1‐SLC4A1 complex was identified with the help of IP.^28^ SLC4A1 could be tightly linked to the spectrin‐based cytoskeleton through binding to specific domains of ANK1.^28^, ^29^ Three mutants in addition to the E142X mutant lost their function as anchoring proteins linked to SPTB and SLC4A1 were determined by Co‐IP. These proteins were retained in ER instead of being localized to the plasma membrane. Therefore, all three mutants were dysfunctional.

Mutations in *ANK1* could contribute to spherocytes with high osmotic fragility, which were observed in the blood of most HS patients.^30^ Here, the HEK293T cell line was used to verify whether the four *ANK1* mutants could increase the osmotic fragility of cells. HEK293T cells are adherent and do not contain haemoglobin; thus, the traditional method, the erythrocyte osmotic fragment test, used in routine clinical examination projects is not applicable. The flow cytometry osmotic fragility test is also used for auxiliary diagnosis of HS,^31^, ^32^ but the result is usually easily affected by the trypsinization for the adherent cells. Higher osmotic fragility results in more easily ruptured cells in the same NaCl solution. Therefore, we explored an improved method for evaluation of osmotic fragility, which is the rate of cell rupture under various hypotonic NaCl solutions with the help of an automated cell counter. All four *ANK1* mutants increased the osmotic fragility of cells according to the method, and the E142X mutant was the most influential because it produces the shortest protein.

Here, we reported two novel mutations in the *ANK1* gene in two unrelated HS Chinese families. We also provided powerful evidence for the pathogenicity of two novel and two previously reported *ANK1* mutations in China. Thus, conducting the same mutation test and providing genetic counselling for the relatives of mutation carriers are meaningful and significant. If other healthy family members at risk have this demand, we can provide pre‐symptomatic gene diagnoses for them. This method will help us to identify asymptomatic mutation carriers and provide diagnosis of HS. For example, in the present study, the two daughters in pedigree 2 carried the same mutation with different clinical phenotypes; therefore, molecular genetic analysis helped to provide diagnosis for the asymptomatic daughters in this family. More generally, the identification of the two new mutations enriches the genetic profile of HS, especially in the Chinese population, which will contribute to the clinical understanding of HS caused by mutations in the *ANK1* gene. Our study also provided an example to test the pathogenicity of *ANK1* mutations found in HS patients by in vivo experiments. Accumulating more mutation data will aid in molecular diagnosis for patients with HS.

## CONFLICT OF INTEREST

The authors declare no conflict of interest.

## AUTHOR CONTRIBUTIONS

LH, JM, DM were in charge of idea, project design and concept of the paper. LH, SC, BZ performed the in vivo experiments. JM, JZ and NJ did experiment of NGS and analysed data. SL and SJ recruited the clinical samples, DNA extraction and clinical assays. LH and DX performed bioinformatic analysis. LH, DM, JM and SJ wrote, edited and revised the manuscript. All authors read and approved the manuscript.
